# Molecular-Scale Analysis of Barium Azide (Ba(N_3_)_2_) Under High Pressure: A Synchrotron X-Ray Diffraction Study on Azide Ion Rotation and Phase Transition

**DOI:** 10.3390/molecules30224426

**Published:** 2025-11-16

**Authors:** Xiaoxin Wu, Wenhui Jiang, Jinwei Zhang, Yuxin Ding, Yang Liu, Yanqing Liu, Hongyang Zhu, Junkai Zhang, Jingshu Wang

**Affiliations:** 1 Key Laboratory of Functional Materials Physics and Chemistry (Ministry of Education), College of Physics, Jilin Normal University, Changchun 130103, China; xiaoxin.wu@hotmail.com (X.W.); jwhxxx02@163.com (W.J.); 13630566338@163.com (J.Z.); d18444099298@126.com (Y.D.); liuyang@jlnu.edu.cn (Y.L.); liuyanqing@jlnu.edu.cn (Y.L.); 2 School of Physics and Electronic Engineering, Linyi University, Linyi 276000, China; 3 Department of Physics and Engineering Physics, The University of Tulsa, Tulsa, OK 74104, USA

**Keywords:** barium azide, high-pressure, phase transition

## Abstract

This study presents a comprehensive investigation of the high-pressure behavior of barium azide Ba(N_3_)_2_ through synchrotron X-ray diffraction, revealing critical insights into its anisotropic compressibility and phase transitions under pressures up to 28 GPa. At ambient conditions, Ba(N_3_)_2_ crystallizes in a monoclinic structure (space group P2_1_/m), exhibiting pronounced anisotropic compression with axial compressibility following the order *b* > *a* > *c*. The distinct compressibility arises from the arrangement of azide ions, where interlayer interactions along the b-axis dominate the response to pressure. A reversible phase transition (Phase I → Phase II) initiates at 2.6 GPa, characterized by a monoclinic-to-monoclinic transformation involving subtle symmetry changes driven by azide ion rotation and lattice plane slippage. Above 11.8 GPa, emergent diffraction peaks suggest a potential secondary transition, though the structure remains stable up to 28 GPa. These findings underscore the unique role of azide ion dynamics in governing structural stability and phase evolution in divalent azides, offering implications for their utility as precursors in polymeric nitrogen synthesis.

## 1. Introduction

To address the growing demand for advanced energetic and nitrogen-rich materials, inorganic azides have emerged as a versatile class of compounds with multifaceted applications spanning industrial, scientific, and technological domains. Industrially, their inherent sensitivity to external stimuli—including heat, light, mechanical shock, and irradiation—renders them indispensable as detonating agents in mining and ordnance, gas generators in automotive airbags (where they rapidly release inert nitrogen to inflate safety systems), and low-temperature pure nitrogen sources for precision synthesis in semiconductor manufacturing and laboratory-scale chemical reactions [1,2]. Beyond practical use, these materials serve as critical theoretical models for probing fundamental phenomena in crystalline solids with complex bonding [3,4], enabling investigations into fast solid-state reactions (e.g., shock-induced decomposition pathways) [5,6], structural stability [7] under extreme conditions, lattice dynamics (such as phonon modes and atomic vibration patterns) [8,9], and electronic structure (including band gaps and charge distribution) [10]. This dual role—practical utility paired with scientific relevance—has sustained long-term interest in understanding their behavior under diverse environments.

In recent decades, a paradigm shift has reshaped the study of inorganic azides: from their traditional role as explosives to their potential as precursors for next-generation energy storage materials, particularly polymeric nitrogen. Polymeric nitrogen, a non-molecular form of nitrogen with extended covalent networks, exhibits extraordinary energy density (exceeding 10 times that of conventional fossil fuels and traditional explosives) and represents a transformative candidate for high-energy-density materials (HEDMs) in aerospace propulsion and advanced batteries [11]. Critically, direct polymerization of molecular nitrogen requires extreme pressures (~200 GPa) and temperatures, which are technologically prohibitive for large-scale applications [12,13]. Inorganic azides circumvent this barrier by enabling polymeric nitrogen formation at substantially lower pressures (~120 GPa for sodium azide, NaN_3_) [14], making them viable platforms for scalable HEDM development. This breakthrough has reignited interest in azide research, with a focus on optimizing their performance as polymeric nitrogen precursors.

Despite significant progress in studying monovalent azides (AN_3_, where A = Li [15], Na [16], K [17], Rb [18], Cs [19], Ag [20], NH^4+^ [21]), which have been extensively characterized under high pressure to elucidate phase transitions, compression mechanisms, and decomposition thresholds, research on divalent azides (B(N_3_)_2_, where B = Mg, Ca [22], Sr [23], Ba, Zn, Mn, Eu, Yb) remains notably limited. Divalent azides hold distinct advantages over their monovalent counterparts for polymeric nitrogen synthesis: their primitive unit cells contain twice the number of nitrogen atoms, theoretically doubling the yield of polymeric nitrogen; they exhibit enhanced structural stability due to the strong ionic bonding characteristic of alkaline-earth metal cations (which mitigates decomposition under high pressure); and their asymmetric azide ion arrangements introduce complex pressure-response behaviors that may unlock novel phase transition pathways [24]. Additionally, while alkaline-earth metal azides are less energetic than some inorganic azides (e.g., lead azide), their energy density surpasses that of alkali metal azides, balancing stability and energy output [25]. For instance, periodic trends in alkali metal azides show that ionic character—and thus chemical reactivity—increases with cation atomic number [26]; extending this trend, barium azide (Ba(N_3_)_2_), with the largest cation radius among alkaline-earth metal azides, is inferred to be more unstable. This heightened instability makes its structure more susceptible to modification under external stimuli such as pressure, making it an ideal candidate for high-pressure studies.

Ba(N_3_)_2_ possesses a distinctive structure among alkali and alkaline-earth metal azides, characterized by asymmetric, nonlinear azide ions and the lowest crystallographic symmetry [27,28]. Consequently, it may exhibit unconventional high-pressure behavior—such as pronounced anisotropic compressibility or distinct phase transition mechanisms—unobserved in other azides. To fill these gaps, this study employs state-of-the-art in situ synchrotron X-ray diffraction (a technique renowned for high resolution and real-time structural monitoring) to systematically investigate Ba(N_3_)_2_ up to 28 GPa. Specifically, we aim to: (1) unravel the mechanisms governing anisotropic compressibility in divalent azides, (2) characterize the nature and driving forces of pressure-induced phase transitions, and (3) establish the relationship between azide ion dynamics (e.g., rotation, bending) and overall structural stability. By addressing these objectives, this work advances fundamental understanding of nitrogen-rich materials and informs the design of next-generation HEDMs and polymeric nitrogen precursors.

## 2. Results and Discussion

**Figure 1:**
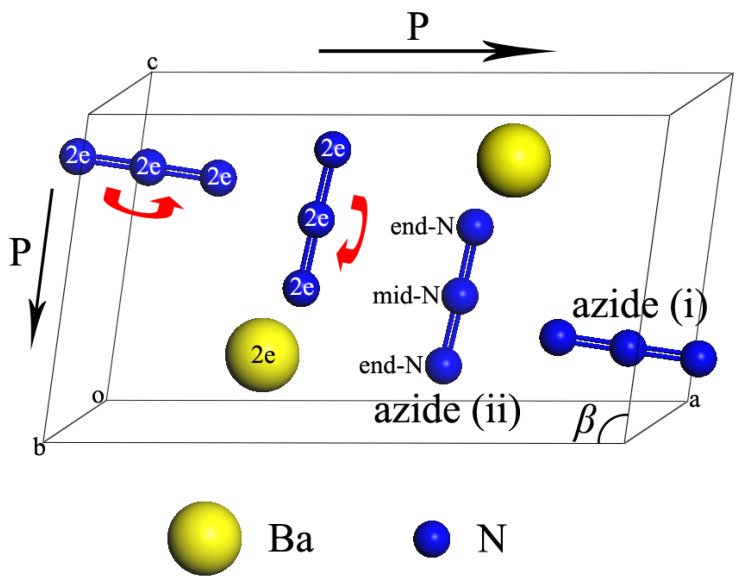
Schematic of the crystal structure of Ba(N_3_)_2_ at ambient conditions. The red colored arrows denote the rotational directions of the azide ions and the black colored arrows denote the slip directions of the (001) planes and the (100) planes under high pressure.

Ba(N_3_)_2_ crystallizes in a monoclinic structure (space group P2_1_/m–C_2h_^2^, Z = 2) at ambient conditions (displayed in Figure 1) [27,29]. The Wyckoff positions of the atoms are all assigned to be 2*e*. Each Ba atom is surrounded by nine azide ions, and the Ba atoms connect with the end-N atoms of azide ions by ionic bonds. The azide ions of Ba(N_3_)_2_ can be classified into two nonequivalent groups, namely azide (i) and azide (ii) (Figure 1). The azide (i) (in the direction nearly parallel to *a* axis) shows a higher symmetrical structure than azide (ii) (in the direction nearly parallel to *c* axis). These two types of azide ions are also approximately perpendicular to each other. As shown in the unit cell of Ba(N_3_)_2_, all atoms lie in two parallel (010) planes, and each plane contains one Ba cation, one azide (i) anion, and one azide (ii) anion.

**Figure 2:**
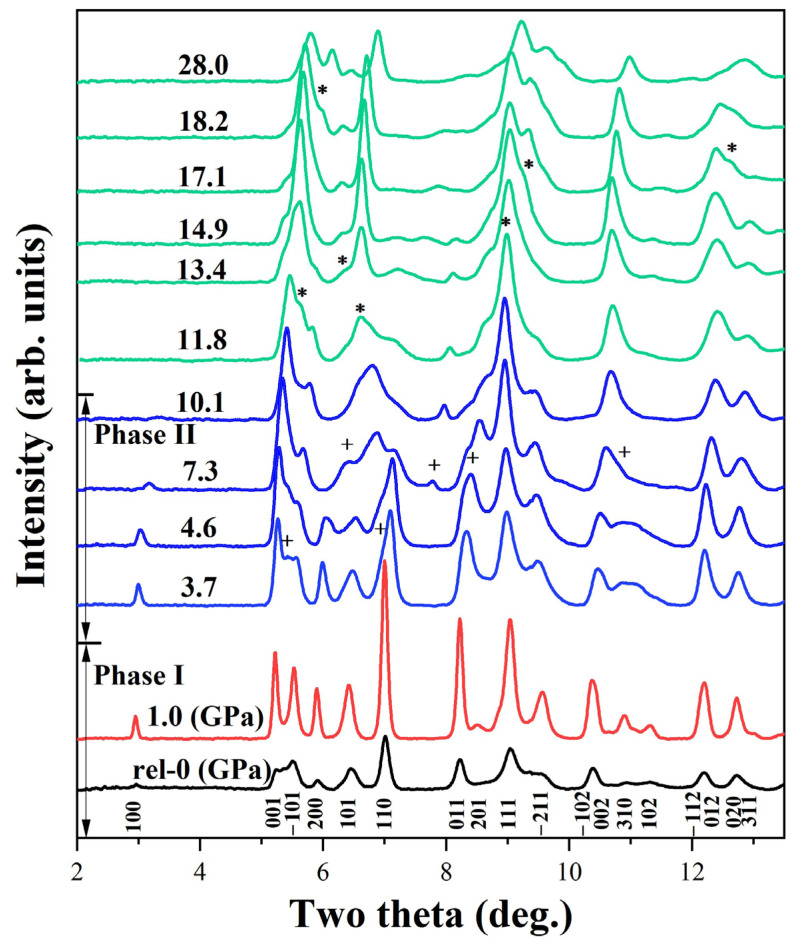
Representative ADXD patterns of Ba(N_3_)_2_ at selected pressures. The pluses (+) and asterisks (*) denote new Bragg diffraction peaks of Ba(N_3_)_2_.

The pressure-dependent structural evolution of Ba(N_3_)_2_ offers critical insights into the behavior of nitrogen-rich materials under extreme conditions, which is essential for designing high-energy-density materials (HEDMs) and polymeric nitrogen precursors, as their compression stability governs energy storage and release. To explore the structural stability of Ba(N_3_)_2_ under extreme conditions, the sample was systematically compressed to 28 GPa. The representative angle-dispersive X-ray diffraction (ADXD) patterns are displayed in Figure 2.

**Figure 3:**
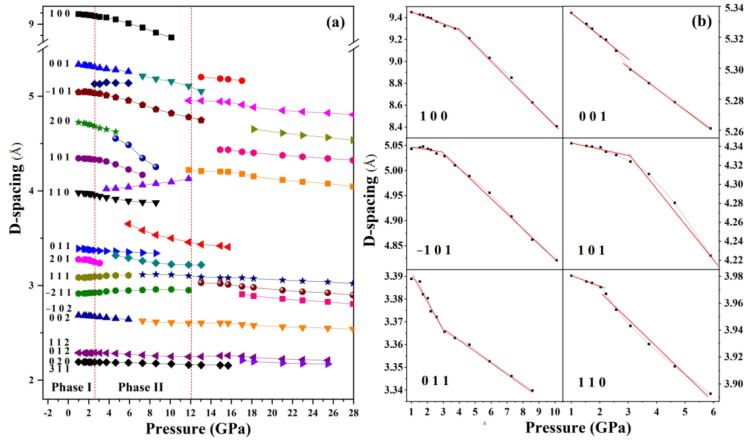
(**a**) D-spacing of Ba(N_3_)_2_ as a function of pressure at room temperature. (**b**) The dependence of D-spacing on pressure of the (100), (001), (−101), (101), (011), (110) planes.

Figure 3 presents the d-spacing of Ba(N_3_)_2_ as a function of pressure at room temperature, along with the quantified lattice contraction for specific crystallographic planes (e.g., (100), (001)). These data provide quantitative evidence that the phase transitions significantly alter the lattice rigidity and compressibility.

**Figure 4:**
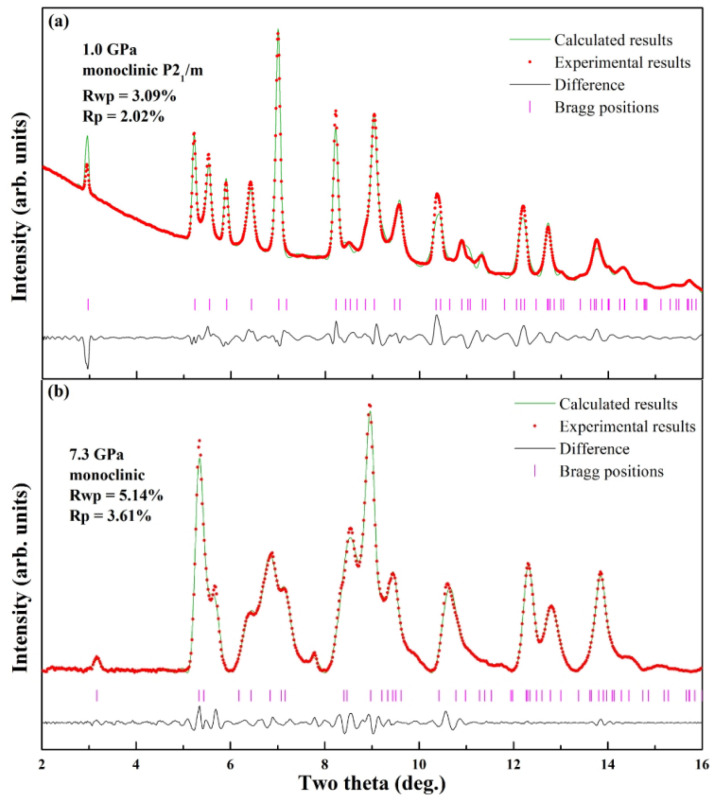
(**a**) Rietveld refinement results of the AXRD patterns of Phase I at 1.0 GPa. (**b**) Pawley refinement results of the AXRD patterns of Phase II at 7.3 GPa.

Two representative pressure points, 1.0 GPa and 7.3 GPa, are selected for detailed structural refinement to characterize the phases as shown in Figure 4. At 1.0 GPa, which lies within the stability field of the ambient-pressure phase (Phase I), Rietveld refinement was successfully applied. All diffraction peaks were fully resolved and indexed without unassigned signals, confirming the anhydrous sample’s high crystallinity and absence of secondary phases (e.g., hydrates or decomposition products). The refined structure conformed to a monoclinic system with space group P2_1_/m and unit cell content Z = 2. Refined lattice parameters *a* = 9.597 Å, *b* = 4.386 Å, *c* = 5.425 Å, *β* = 99.98°, and *V*_0_ = 224.90 Å^3^, which are in good agreement with the previous literature [27]. In contrast, for the high-pressure Phase (Phase II) at 7.3 GPa—a pressure well within its stability range—the specific space group could not be determined unambiguously from the available data. Therefore, Pawley refinement was employed to analyze the ADXD pattern. This approach yielded excellent reliability factors (Rwp = 5.14%, Rp = 3.61%), confirming that the diffraction data are consistent with a monoclinic lattice and providing reliable unit cell parameters for this high-pressure phase, even in the absence of a full structural model.

**Figure 5:**
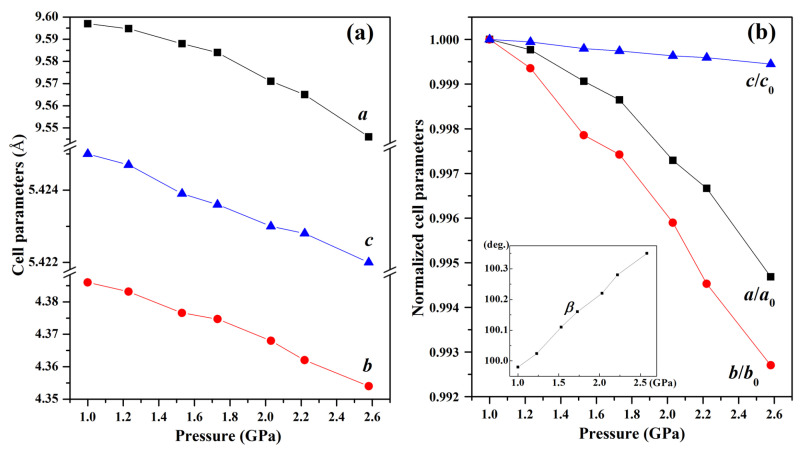
(**a**) Pressure dependence of the cell parameters *a*, *b*, and *c* of the ambient pressure phase Ba(N_3_)_2_. (**b**) Pressure dependence of the compression ratios *a*/*a*_0_, *b*/*b*_0_, and *c*/*c*_0_ and the monoclinic angle *β* of the ambient pressure phase Ba(N_3_)_2_.

During the compression process, Phase I remains stable up to 2.6 GPa, as evidenced by the absence of any detectable changes in diffraction peak positions, intensities, or widths. This indicates that the Phase I lattice can withstand moderate compression without undergoing structural rearrangement under 2.6 GPa. The anisotropic contraction across different planes suggests a structural response where the lattice softens along specific directions prior to the transition, lowering the energy barrier for the subsequent phase transformation. Figure 5a demonstrates distinct compression behaviors along different crystallographic axes within the pressure region of 1.0~2.6 GPa. The *b*-axis (99.27% compression) shows significantly greater compressibility than *a*-axis (99.47%) and *c*-axis (99.95%). This anisotropic response originates from the crystallographic arrangement of azide ions—while the *b*-axis compression is facilitated by weak interlayer interactions between (010) planes, the *a*-axis resistance stems from strong electrostatic repulsions between adjacent azide (i) anions. Notably, the *c*-axis exhibits intermediate behavior due to competing effects: the shorter inter-azide distance (5.343 Å) between azide (ii) ions creates inherent resistance, yet the mixed ionic-covalent character allows partial stress dissipation through bond angle adjustments.

Based on the observed anisotropic compressibility and the evolution of the monoclinic angle *β*, we propose that the primary mechanism driving the Phase I → Phase II transition involves the differential rotation of azide ions, facilitated by lattice plane slippage. The rotational dynamics of azide ions under pressure—visually indicated by red arrows in Figure 1—are proposed to serve as a key mechanism for the phase transition of the Phase I → Phase II transition at 2.6 GPa, bridging molecular-level behavior to macroscale structural changes. A critical observation is the differential rotation rates between azide (i) and azide (ii) species: azide (i), with its higher symmetry (near-parallel to the *a*-axis and more uniform N-N bond lengths), exhibits faster rotational motion compared to the lower-symmetry azide (ii) (aligned with the *c*-axis). This disparity directly correlates with the anisotropic axial compressibility observed: faster rotation of azide (i) reduces steric hindrance along the *a*-axis, facilitating greater compression of this axis relative to the *c*-axis, where slower-rotating azide (ii) maintains more rigid lattice packing.

This molecular rotation is not arbitrary but driven by crystallographic plane slippage, as explicitly supported by the pressure-induced variation in the monoclinic angle *β* (inset of Figure 5b). As pressure increases toward 2.6 GPa, *β* gradually deviates from its ambient value (99.98°), a change attributed to the gliding of (100) and (001) lattice planes (marked by black arrows in Figure 1). These plane movements generate tangential forces that act as torque on adjacent azide ions, initiating their rotation. This creates a novel stress-accommodation mechanism: molecular reorientation occurs first to relieve local lattice stress, followed by subtle structural adjustments—rather than immediate bond breaking, which is the primary response assumed in conventional high-pressure phase transition models. This sequence challenges traditional frameworks by highlighting that nitrogen-rich materials can adapt to pressure via coordinated molecular rearrangements before large-scale lattice reconstruction. While the data presented here most consistently supports the model of azide ion rotation, we cannot rule out the potential contribution of other subtle structural adjustments, such as changes in electron density distribution or minor bond-length modifications, which may work in concert with the primary rotational mechanism.

However, with further compression beyond 2.6 GPa, distinct changes in the X-ray diffraction patterns began to emerge. As evidenced by the data in Figure 2 and Figure 3, the structural phase transition is marked by the gradual attenuation of characteristic Phase I peaks, the emergence of new diffraction features, and subtle shifts in peak positions. This newly formed Phase II. A notable feature of this transition is that several diffraction peaks of Phase I were retained in the patterns of Phase II, suggesting a degree of structural continuity between the two phases rather than a complete breakdown and reconstruction of the crystal lattice. Nevertheless, a significant alteration in compressibility is evidenced by the substantially changed slopes of the d-spacing versus pressure curves for these retained peaks after the phase transition, reflecting different mechanical responses of the corresponding lattice planes in Phase II. This change in compressibility is a direct consequence of the structural adjustments that occur during the transition. In addition, almost all the new diffraction peaks of Phase II (labeled with pluses (+) in Figure 3) can be traced to the splitting of existing peaks of Phase I—a phenomenon typically associated with structural distortions (e.g., changes in lattice symmetry or atomic coordination environments) rather than the formation of an entirely new crystal system.

On the basis of these observations, this Phase I → Phase II transition is most likely a monoclinic-to-monoclinic transformation involving subtle symmetry changes. This conclusion is strongly supported by the fact that the structure of Phase II could be reliably indexed into a monoclinic lattice. To further validate this structural assignment, Pawley refinement of the ADXD pattern of Phase II acquired at 7.3 GPa (a pressure well within the stability range of Phase II), the results are illustrated in Figure 4b, confirming that Phase II is reasonably described as a distorted variant of Phase I. Importantly, this distortion preserves the bonding characters between Ba^2+^ cations and azide (N) anions—specifically, the ionic interactions between Ba^2+^ and the terminal N atoms of N_3_^−^ ions remain intact, which explains the retention of some Phase I peaks in Phase II.

Furthermore, by analyzing the trend of changing atomic positions in Phase I as pressure increases, it can be inferred that the phase transition is likely induced by the rotation and bending behavior of the azide ions. In azide compounds, such rotational and bending motions of N_3_^−^ ions are common responses to high pressure, as they allow the lattice to accommodate compression by adjusting the spatial arrangement of the anions without disrupting the overall cation sublattice. Critically, displacive phase transitions—those involving only symmetrical conformational changes (e.g., rotations, bending) of ions rather than the diffusion or rearrangement of atoms into new lattice sites. Given that the bonding environment of Ba^2+^ ions is preserved and the transition involves peak splitting (not a change in crystal system), the transition between Phase I and Phase II is therefore probably a subtle symmetry change within the monoclinic system.

All experimental evidence converges to the conclusion that Phase II is consistent with a monoclinic structure, with unit cell parameters that differ slightly from those of Phase I (primarily in the monoclinic angle *β* and the relative dimensions of the lattice axes). This subtle symmetry change, driven by the distortion of the lattice and the reorientation of azide ions, represents a key adaptation of Ba(N_3_)_2_ to high-pressure conditions, ensuring the structural stability as pressure increases beyond 2.6 GPa. However, based on the current synchrotron X-ray diffraction data we have obtained, we are unable to perform a full structural refinement to determine the atomic fractional coordinates of Phase II. The primary challenge lies in the complexity of the structure and the relatively limited number of observable diffraction peaks under high pressure, which makes it difficult to reliably refine the positions of light atoms like nitrogen. Consequently, our Pawley refinement successfully determined the unit cell parameters of Phase II, confirming its monoclinic symmetry and the changes in lattice metrics, but it does not provide the precise atomic arrangements needed to directly illustrate the azide rotations.

Phase II of Ba(N_3_)_2_ remains structurally stable up to 10.1 GPa, with no detectable changes in its diffraction peak positions or intensities within this pressure range. As pressure increases further, several new diffraction peaks start to emerge at 11.8 GPa (labeled with asterisks (*) in Figure 2), a clear sign that may indicate the onset of another phase transition (Phase II → Phase III). However, fully identifying the details of this transition proves challenging. A key barrier is the uncertain atomic fractional coordinates of Phase II—without precise knowledge of where Ba^2+^ cations and N_3_^−^ anions are situated in Phase II, modeling the atomic rearrangement into a new phase becomes difficult. Additionally, the structural complexity of N atoms, such as potential variations in N_3_^−^ ion geometry or subtle N-N interactions, complicates the interpretation of diffraction data. Thus, further studies combining advanced experiments and theoretical calculations are necessary to characterize this transition. The sample was ultimately compressed to 28.0 GPa, with no additional transformations observed.

The reversibility of the pressure-induced phase transitions was unequivocally demonstrated by the complete recovery of the original Phase I structure after decompression from 28 GPa to ambient conditions. As shown in Figure 1, the X-ray diffraction pattern of the recovered sample exhibits perfect alignment in peak positions with the reference Phase I pattern collected at 1.0 GPa. This precise recovery of all characteristic diffraction peaks confirms that the series of structural transformations—from Phase I to Phase II and potentially to Phase III—is fully reversible and does not involve irreversible chemical decomposition or bond breaking. Notably, while the peak positions are perfectly restored, a slight reduction in diffraction intensity and minor peak broadening are observed in the recovered sample. These features are characteristic of the development of microstrains and crystallographic defects introduced during the complex cycle of multiple phase transitions and lattice rearrangements under extreme compression. Such microstructural changes are commonly observed in high-pressure studies of crystalline materials and do not detract from the fundamental conclusion of structural reversibility. Rather, they provide insight into the kinetic barriers and metastable states involved in the phase transition pathways.

The chemical integrity and compositional stability of Ba(N_3_)_2_ throughout the compression-decompression cycle were robustly confirmed by our in situ X-ray diffraction data. The absence of any new diffraction peaks attributable to decomposition products (such as elemental barium or barium nitride) or impurities in all patterns up to 28 GPa (Figure 2) provides primary evidence that no chemical decomposition occurred under high pressure. Furthermore, the complete reversibility of the diffraction pattern to the original Phase I structure upon decompression offers conclusive proof against irreversible chemical changes. The preservation of sharp diffraction peaks throughout the entire pressure cycle also rules out significant amorphization or degradation. Therefore, the collective evidence from XRD is considered definitive for demonstrating the constancy of the sample’s composition under high pressure.

These findings hold significant implications for both fundamental and applied materials science. Fundamentally, the high-pressure behavior of Ba(N_3_)_2_ offers new insights into how nitrogen-rich materials adapt to extreme pressure—especially the observation of an subtle symmetry change within the monoclinic system driven by azide rotation rather than symmetry breaking. This challenges traditional models of high-pressure transformations in inorganic compounds and provides new design principles for materials with complex anion arrangements. Technologically, the stability of Ba(N_3_)_2_ under compression and high nitrogen density make it a promising candidate for next-generation energy storage. Its reversible phase transitions without decomposition also open paths to safer, more controllable high-energy-density materials compared to traditional explosives, while its anisotropic compressibility could guide the design of layered materials with tailored energy release profiles.

## 3. Materials and Methods

We purchased barium azide monohydrate (Ba(N_3_)_2_·H_2_O) from Shanghai Zhaozhi nano technology Co. (Shanghai, China). White anhydrous barium azide (Ba(N_3_)_2_) powder was obtained by heating the monohydrate precursor at 120 °C under a controlled atmosphere. The phase purity and chemical integrity of the anhydrous product were confirmed by X-ray diffraction, which showed a perfect match with the known monoclinic structure (space group P2_1_/m) and no detectable Bragg peaks corresponding to barium hydroxide, oxide, or other decomposition products.

High-pressure experiments were performed in a symmetric diamond anvil cell with a flat anvil of 500 μm in diameter. T301 steel sheets were used as gaskets with a sample chamber of 100 μm in diameter and 50 μm in thickness. The pressures were measured by the ruby fluorescence technique [30]. A mixture of methanol and ethanol with a volume ratio of 4:1 was chosen as the pressure-transmitting medium of the high pressure synchrotron X-ray study. The high-pressure synchrotron angle-dispersive X-ray diffraction measurements were performed using an angle-dispersive synchrotron X-ray source (0.485946 Å) at the B2 High-Pressure Station of Cornell High Energy Synchrotron Source (CHESS). The Bragg diffraction rings were recorded with a MAR165 image plate detector(by MAR USA, Inc., Evanston, IL, USA). The images were converted to 2-theta versus intensity using FIT2D software (v12). Then, Material Studio Software (v5.5) was utilized to refine the structure and analyze the structural evolution using the Rietveld and Pawley methods, respectively.

## 4. Conclusions

This study provides comprehensive insights into the high-pressure behavior of barium azide (Ba(N_3_)_2_) through synchrotron X-ray diffraction analysis up to 28 GPa. The results reveal that Ba(N_3_)_2_ exhibits pronounced anisotropic compressibility (*b* > *a* > *c*), governed by interlayer interactions along the *b*-axis and strong repulsions between azide (i) anions along the *a*-axis. A reversible subtle symmetry change occurs at 2.6 GPa, characterized by azide ion rotation and lattice plane slippage while maintaining monoclinic symmetry. Notably, this transition mechanism challenges conventional models of high-pressure transformations in nitrogen-rich materials, as it involves coordinated molecular rearrangements that fine-tune the crystal symmetry without breaking the monoclinic structure. Above 11.8 GPa, emergent diffraction peaks suggest a potential secondary transition, though the structure remains stable up to 28 GPa without decomposition. The material’s exceptional stability and reversible phase transitions highlight its potential as a precursor for polymeric nitrogen synthesis and energy storage applications. These findings not only advance our understanding of divalent azides under extreme conditions but also establish design principles for developing next-generation high-energy-density materials.

## Data Availability

The original contributions presented in this study are included in the article. Further inquiries can be directed to the corresponding authors.
